# High Production Rates Sustain *In Vivo* Levels of PD-1^high^ Simian Immunodeficiency Virus-Specific CD8 T Cells in the Face of Rapid Clearance

**DOI:** 10.1128/JVI.01001-13

**Published:** 2013-09

**Authors:** Constantinos Petrovas, Takuya Yamamoto, David A. Price, Srinivas S. Rao, Nichole R. Klatt, Jason M. Brenchley, Daniel C. Douek, Emma Gostick, Bastian R. Angermann, Zvi Grossman, Derek C. Macallan, Martin Meier-Schellersheim, Richard A. Koup

**Affiliations:** Immunology Laboratorya; Human Immunology Sectionb; Laboratory of Animal Medicine,d Vaccine Research Center, National Institute of Allergy and Infectious Diseases, National Institutes of Health, Bethesda, Maryland, USA; Institute of Infection and Immunity, Cardiff University School of Medicine, Cardiff, United Kingdomc; Laboratory of Molecular Microbiology, National Institute of Allergy and Infectious Diseases, National Institutes of Health, Bethesda, Maryland, USAe; Program in Systems Immunology and Infectious Disease Modeling, Laboratory of Immunology, National Institute of Allergy and Infectious Diseases, National Institutes of Health, Bethesda, Maryland, USAf; Tel Aviv University School of Medicine, Tel Aviv, Israelg; Laboratory of Immunology, National Institute of Allergy and Infectious Diseases, National Institutes of Health, Bethesda, Maryland, USAh; Infection and Immunity Research Centre, Division of Clinical Sciences, St. George's Hospital, University of London, London, United Kingdomi

## Abstract

Programmed Death 1 (PD-1) expression by human/simian immunodeficiency virus (HIV/SIV)-specific CD8 T cells has been associated with defective cytokine production and reduced *in vitro* proliferation capacity. However, the cellular mechanisms that sustain PD-1^high^ virus-specific CD8 T cell responses during chronic infection are unknown. Here, we show that the PD-1^high^ phenotype is associated with accelerated *in vivo* CD8 T cell turnover in SIV-infected rhesus macaques, especially within the SIV-specific CD8 T cell pool. Mathematical modeling of 5-bromo-2′ deoxyuridine (BrdU) labeling dynamics demonstrated a significantly increased generation rate of PD-1^high^ compared to PD-1^low^ CD8 T cells in all memory compartments. Simultaneous analysis of Ki67 and BrdU kinetics revealed a complex *in vivo* turnover profile whereby only a small fraction of PD-1^high^ cells, but virtually all PD-1^low^ cells, returned to rest after activation. Similar kinetics operated in both chronic and acute SIV infection. Our data suggest that the persistence of PD-1^high^ SIV-specific CD8 T cells in chronic infection is maintained *in vivo* by a mechanism involving high production coupled with a high disappearance rate.

## INTRODUCTION

The expression of Programmed Death 1 (PD-1) has been linked to CD8 T cell dysfunction, especially defective cytokine production and impaired *in vitro* proliferation capacity, in human/simian immunodeficiency virus (HIV/SIV) infection (1–5). Chronic T cell receptor (TCR)-mediated stimulation is necessary to maintain high PD-1 expression on SIV-specific CD8 T cells (6) and, most likely, on HIV-specific CD8 T cells (7). Furthermore, PD-1 expression correlates with reduced antigen-specific CD8 T cell survival *in vitro* (8–10). However, it is not known whether PD-1^+^ CD8 T cell survival is compromised *in vivo*.

Several studies have described *in vivo* T cell dynamics during HIV and SIV infection (11–17). It is clear from this body of work that both HIV and SIV infection lead to increased turnover of CD4 and CD8 T cells *in vivo*. Although the interpretation of labeling studies that focused on CD4 T cell kinetics was confounded by the fact that these cells are the major targets for cytopathic viral infection, it became clear that increased turnover of CD4 and CD8 T cells alike is primarily a physiological consequence of incessant immune activation, both specific and nonspecific (18). The study of CD8 T cell dynamics is therefore important, not only in its own right, but also as a paradigm for understanding the consequences of ongoing immune activation.

It is unclear how a large population of PD-1^+^ antigen-specific CD8 T cells is maintained in the presence of continuous HIV/SIV replication, especially when all data suggest a critical role for chronic antigen stimulation, which should also induce apoptosis. One possibility is that PD-1^+^ virus-specific CD8 T cells are maintained via slow production and slow clearance, suggesting that they do not actually undergo apoptosis in response to *in vivo* stimulation as they do *in vitro*, perhaps due to an altered cytokine environment. Alternatively, these cells could persist in a more dynamic state of high production and high clearance, constantly replenished from a population of PD-1^−^ virus-specific CD8 T cells. To distinguish between these two possibilities, we investigated the turnover of bulk and SIV-specific CD8 T cell populations by analyzing the cellular incorporation of 5-bromo-2′deoxyuridine (BrdU) in SIV-infected rhesus macaques.

## MATERIALS AND METHODS

### Animals.

Four colony-bred rhesus macaques (Covance Research Products), housed and handled in accordance with the standards of the American Association for the Accreditation of Laboratory Animal Care, were infected intravenously (i.v.) with SIVmac251. All animal studies were approved by the Animal Care and Use Committees of the Vaccine Research Center, National Institute of Allergy and Infectious Diseases, NIH. Viral loads were measured using a QIAamp Viral RNA Mini Kit (Qiagen) at Duke Human Vaccine Institute, Durham, NC. Peripheral blood mononuclear cells (PBMCs) were isolated from whole blood by centrifugation over an isotonic discontinuous Percoll (Sigma) density gradient (35% to 60% [vol/vol]). After washing, the cells were cryopreserved until further use.

### Antibodies.

Directly conjugated monoclonal antibodies (MAbs) and other fluorescent reagents were obtained from the following vendors: (i) αCD3-Cy7APC (allophycocyanin), αCD95-Cy5PE (phycoerythrin), αBrdU-APC, and αKi67-FITC (fluorescein isothiocyanate) from BD Biosciences; (ii) αCD8-QD655, streptavidin-Cy7PE, fixable violet amine viability dye (ViViD), and quantum dots from Life Technologies; and (iii) αCD28-ECD (energy-coupled dye) from Beckman Coulter. Polyclonal αPD-1–biotin was obtained from R&D Systems. The fluorescent-peptide–Mamu-A*01 tetrameric complexes central memory 9 (CM9)-PE (Gag, CTPYDINQM; residues 181 to 189) and TL8-PE (Tat, TTPESANL; residues 28 to 35) were produced in house, as described previously (19).

### Flow cytometry.

Briefly, 2 × 10^6^ to 3 × 10^6^ cells were washed and stained sequentially with ViViD, αPD-1–biotin, and fluorochrome-labeled streptavidin. After two further washes, the cells were incubated with tetramer at 37°C for 20 min, washed twice, and surface stained with αCD8, αCD28, and αCD95 for 20 min, and then fixed with 1× fluorescence-activated cell sorter (FACS) lysing solution (BD Biosciences) and permeabilized with a Cytofix/Cytoperm Kit (BD Biosciences). The cells were then treated with DNase (APC BrdU Flow Kit; BD Biosciences) for 30 min at 37°C, washed with Perm/Wash buffer (Cytofix/Cytoperm Kit; BD Biosciences), and stained intracellularly with αCD3, αBrdU, and αKi67. The gating scheme is shown in Fig. S1A in the supplemental material. The CD28^dim^ CD95^low^ (here referred to as naive) CD8 T cell population was used to define PD-1^high^ and PD-1^low^ expression. Cells were analyzed using a modified LSRII flow cytometer (BD Immunocytometry Systems). Between 500,000 and 10^6^ events were acquired for each condition. Antibody capture beads (BD Biosciences) stained separately with the individual MAbs used in the test samples were used for electronic compensation. Data analysis was performed using FlowJo version 9.0.1 (TreeStar). The forward scatter area (FSC-A) versus forward scatter height (FSC-H) profile was used to gate out cell aggregates; ViViD was used to exclude apoptotic cells. After selection based on CD3 positivity, BrdU and Ki67 expression was measured in gated CD4, CD8, and tetramer^+^ CD8 T cells with respect to differentiation status and PD-1 expression.

### *In vivo* administration of BrdU.

BrdU (Sigma) was dissolved in Hanks balanced salt solution (Life Technologies) at 10 mg/ml, pH 7.4, and sterile filtered into autoclaved bottles. Macaques received 30 mg/kg body weight of BrdU daily via i.v. injection on four consecutive days. Blood was collected on days 1 (pre-BrdU injection, basal level), 2, 3, 4, 7, 9, and 14 for acute-phase studies; blood samples were collected on days 22 and 31, in addition, for chronic-phase studies. The same macaques were studied in both acute- and chronic-phase SIV infection using separate courses of BrdU administration.

### Mathematical model.

A system of coupled ordinary differential equations (ODE) was defined to explore the kinetic behavior of three different cell states defined as resting (*R*), activated (*A*), and effector (*E*). In this model, resting cells can become activated at rate *a*, and activated cells can divide at rate *p* or differentiate (at rate *e*) into effectors that can disappear at rate *d* or return to rest (state *R*) at rate *r*. The differential equations for the time evolution of the population sizes are as follows: (*d*/*dt*)*R* = *r* × *E* − *a* × *R*, (*d*/*dt*)*A* = *a* × *R* + (*p* − *e*) × *A*, and (*d*/*dt*)*E* = *e* × *A* − (*d* + *r*) × *E*.

The BrdU labeling and delabeling process was simulated by assuming that during the labeling phase, cells that divided became BrdU^high^, whereas during the delabeling phase, cells that divided lost BrdU on average in four steps, meaning that four divisions were necessary to transition from BrdU^high^ to an unlabeled state. This “minimal model” simplifies the dilution process because the BrdU population generated during the labeling period is heterogeneous in terms of labeling intensity; thus, some labeled cells that continue to divide become negative earlier than others. Mathematically, this was captured by using five subgroups of *R*, *A*, and *E*, corresponding to four BrdU^+^ groups and one BrdU^−^ group. The parameters *r*, *a*, *e*, *p*, and *d* of the model were fitted against the experimental data (fractions of BrdU^+^ cells, total and Ki67^−^, in blood over time) using the above ODEs in MATLAB (details can be found in the supplemental material). Steady state was assumed and confirmed experimentally (see Fig. S1B in the supplemental material).

### Statistical analysis.

All statistical analyses were performed using GraphPad Prism (GraphPad Software). *P* values were calculated using the Mann-Whitney *U* test, and values of <0.05 were considered significant. The mean values ± standard errors are also presented. BrdU decay rates were calculated assuming first-order kinetics. The natural log of the percentage of BrdU^high^ CD8 T cells was plotted against time. These data were fitted to a straight line using the method of least squares and the first-order rate constant determined from the slope of the line.

## RESULTS

### High turnover of CM9^+^ PD-1^high^ CD8 T cells during the chronic phase of SIV infection.

**Fig 1:**
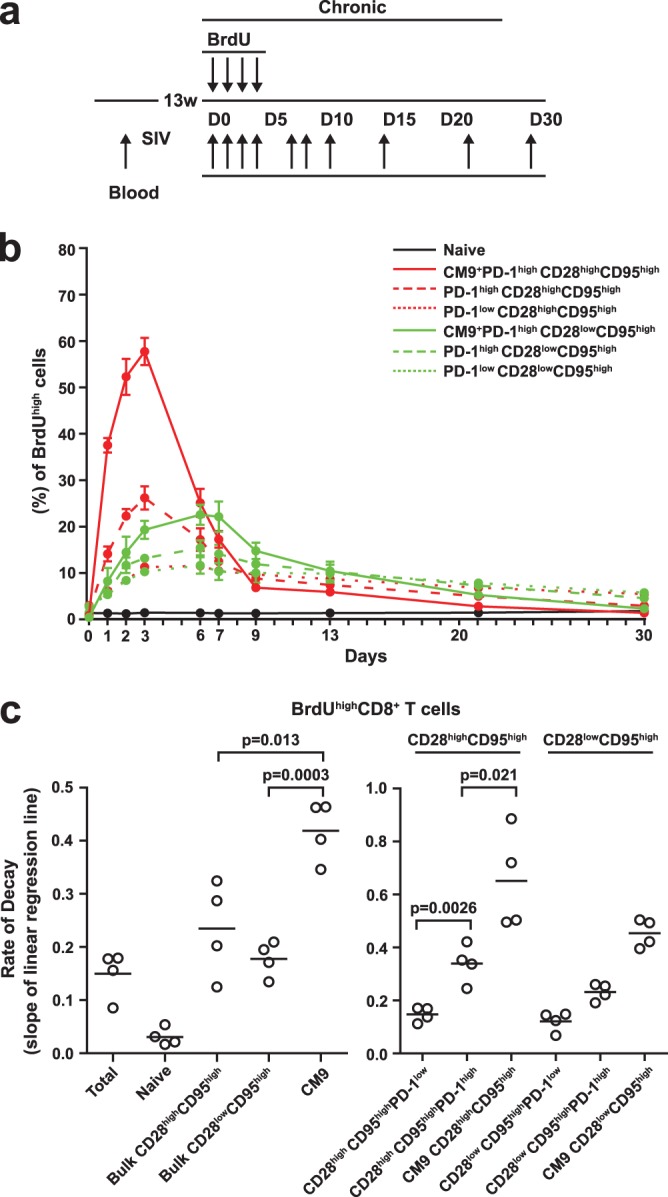
PD-1^high^ SIV-specific CD8 T cells are characterized by high *in vivo* turnover. (a) Schematic representation of BrdU administration and blood collection during the chronic phase of SIV infection. D, day; w, week. (b) BrdU kinetics in CM9^+^ and bulk memory CD8 T cells. The error bars indicate standard errors. (c) Dot plots depicting BrdU decay rates in naive, bulk memory, and CM9^+^ CD8 T cell populations (left) with stratification for PD-1 expression (right). Decay rates were calculated assuming first-order kinetics. The horizontal bars indicate mean values. *P* values were calculated using the Mann-Whitney *U* test.

To investigate the *in vivo* dynamics of rigorously defined T cell populations in rhesus macaques 3 to 4 months after SIVmac251 infection, we conducted a serial analysis of BrdU incorporation using flow cytometry and mathematical modeling (Fig. 1a). No BrdU integration was detected in naive CD8 T cells, indicating very slow *in vivo* turnover within the compartment (Fig. 1b). In agreement with previously published data (6), almost all CM9-specific CD8 T cells were found to express a PD-1^high^ phenotype (see Fig. S1A and B in the supplemental material). BrdU incorporation was higher in the CD28^high^ CD95^high^ population of CM9^+^ PD-1^high^ CD8 T cells than in the corresponding CD28^low^ CD95^high^ population (57.7 ± 2.9 versus 22.5 ± 2.1, maximum percentage of BrdU^high^ cells; *P* = 0.0294) (Fig. 1b). The accumulation of BrdU was also accelerated in the CM9^+^ PD-1^high^ CD28^high^ CD95^high^ population (peak on day 3 or 4) compared to the CM9^+^ PD-1^high^ CD28^low^ CD95^high^ population (peak on day 5 or 6) (Fig. 1b; see Fig. S2 in the supplemental material). Additionally, CM9^+^ CD8 T cells incorporated significantly larger amounts of BrdU than matched bulk CD8 T cells in both the CD28^high^ CD95^high^ (26.2 ± 2.4, maximum percentage of BrdU^high^ cells) and CD28^low^ CD95^high^ (15.4 ± 1.5, maximum percentage of BrdU^high^ cells) populations. Both CM9^+^ and bulk CD8 T cell populations, however, exhibited similar kinetics of BrdU incorporation (Fig. 1b). The frequency of TL8^+^ SIV-specific CD8 T cells during the chronic phase was very low (see Fig. S1B in the supplemental material), making the analysis of BrdU incorporation problematic. Analysis of bulk memory CD8 T cell subsets revealed higher *in vivo* incorporation of BrdU in the PD-1^high^ CD28^high^ CD95^high^ population than in the PD-1^high^ CD28^low^ CD95^high^ population (26.2 ± 2.4 versus 15.4 ± 1.5, maximum percentage of BrdU^high^ cells; *P* = 0.0286) (Fig. 1b). Similarly to CM9^+^ CD8 T cells, the kinetics of BrdU incorporation differed between these two populations; BrdU accumulation peaked at day 3 or 4 for the PD-1^high^ CD28^high^ CD95^high^ population, while the peak for PD-1^high^ CD28^low^ CD95^high^ cells occurred on day 5 or 6. PD-1^low^ cells were characterized by lower *in vivo* incorporation of BrdU than PD-1^high^ cells in both memory compartments (Fig. 1b; see Fig. S2 in the supplemental material).

BrdU decay rates were calculated assuming first-order kinetics. As expected, no decay was apparent in the naive population (Fig. 1c, left). The decay rate was significantly higher in CM9^+^ cells (0.418 ± 0.028 per day) than in the bulk CD28^high^ CD95^high^ (0.234 ± 0.044 per day; *P* = 0.013) and CD28^low^ CD95^high^ (0.177 ± 0.016 per day; *P* = 0.0003) CD8 T cell populations (Fig. 1c, left). We further analyzed BrdU decay rates in individual memory populations with respect to PD-1 levels (Fig. 1c, right). Again, significantly higher decay rates were found for CM9^+^ cells than for bulk PD-1^high^ CD8 T cells in both memory populations (0.651 ± 0.09 versus 0.338 ± 0.032 per day, CD28^high^ CD95^high^, *P* = 0.021; 0.453 ± 0.02 versus 0.231 ± 0.015 per day, CD28^low^ CD95^high^, *P* = 0.0004). Furthermore, a trend toward higher decay rates in CM9^+^ CD28^high^ CD95^high^ cells than in CM9^+^ CD28^low^ CD95^high^ cells was observed (Fig. 1c, right). Interestingly, bulk PD-1^high^ CD8 T cells were characterized by significantly higher BrdU decay rates than for PD-1^low^ CD8 T cells in both memory compartments (0.338 ± 0.032 versus 0.147 ± 0.014 per day, CD28^high^ CD95^high^, *P* = 0.0026; 0.231 ± 0.015 versus 0.121 ± 0.018 per day, CD28^low^ CD95^high^, *P* = 0.004) (Fig. 1c, right). Overall, our data point to accelerated *in vivo* turnover of CM9^+^ PD-1^high^ CD8 T cells and indicate that PD-1^low^ CD8 T cells have the lowest turnover among the populations tested.

### CM9^+^ PD-1^high^ CD8 T cells are characterized by high *in vivo* generation rates.

**Fig 2:**
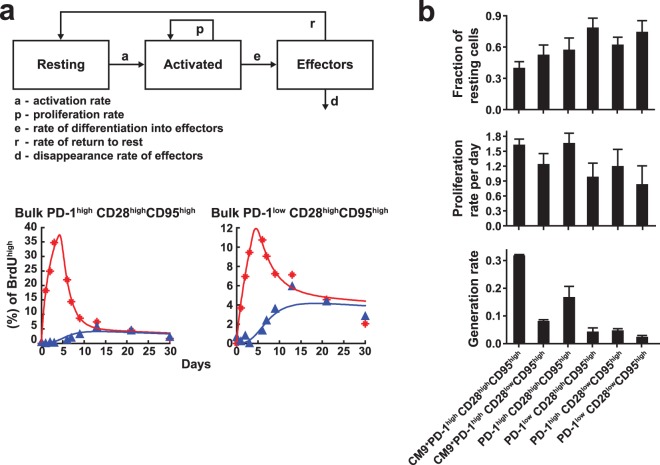
PD-1^high^ SIV-specific CD8 T cells are characterized by high *in vivo* generation rates. (a) Graphic overview of the model used to analyze specific populations (top) and representative fitting curves for BrdU levels in the indicated cell populations from one macaque (bottom). The red data points and curve correspond to the total BrdU uptake; the blue data points and curve represent only the Ki67^−^ BrdU^+^ cells. (b) Calculated fractions of resting cells, proliferation rates, and generation rates, calculated as the product of the fraction of activated cells and the proliferation rate, in the various phenotypically distinct populations. The bars depict mean values plus standard errors.

Despite the high *in vivo* turnover of CM9^+^ CD8 T cells, the percentage (∼0.7%) of these cells in the periphery remained relatively constant over the 31 days of observation (see Fig. S1B in the supplemental material). Analysis of PD-1 and Ki67 expression revealed a similar steady state for all populations tested (see Fig. S1B in the supplemental material). These are important prerequisites for the modeling approach used here, which is therefore not well suited to acute-phase analyses. To explore the proliferation and disappearance kinetics of the various populations more thoroughly, we analyzed *in vivo* turnover using a simple five-parameter ODE model (Fig. 2a). Due to the limited number of data points, we opted for a simple model that nevertheless accounts for the fact that activated cells will first proliferate and then contract (differentiate, die, and disappear). A simpler model with only four parameters, according to which cells were assumed to proliferate and disappear simultaneously, was able to fit the observed total BrdU uptake and BrdU loss kinetics for most cell populations but failed to reproduce the kinetics of the appearance of Ki67^−^ BrdU^+^ cells (Fig. 2a, bottom); this observation emphasizes the importance of taking into account the time-structured “cohort” behavior of activated cells (18, 20). A representative “fitting curve,” generated by fitting the parameters of this model to one of our data sets, is shown in Fig. 2a (bottom). The complete set of fits is provided in Fig. S2 in the supplemental material. CM9^+^ CD8 T cells were characterized by higher activation/proliferation rates than bulk CD8 T cells in both the CD28^high^ CD95^high^ and CD28^low^ CD95^high^ compartments (Fig. 2b). The generation rate was calculated based on the product of the proliferation rate and the fraction of activated cells in each particular population. CM9^+^ CD8 T cells had a significantly increased generation rate compared to PD-1^high^ bulk memory (CD28^high^ CD95^high^ and CD28^low^ CD95^high^) CD8 T cells (Fig. 2b). Cells with low PD-1 expression had the lowest generation rates in both memory populations. A striking hierarchy was also apparent with respect to the fraction of resting cells. In particular, CM9^+^ CD28^high^ CD95^high^ CD8 T cells displayed the smallest fraction of cells in the resting state (40%), while substantially larger fractions were observed in the bulk memory populations, especially those with low levels of PD-1 expression. Taken together, our data indicate that a high-generation/high-disappearance mechanism underlies the maintenance of antigen-specific and bulk PD-1^high^ CD8 T cell populations in chronic SIV infection.

### Only a small fraction of activated PD-1^high^ CD8 T cells return to a resting state.

**Fig 3:**
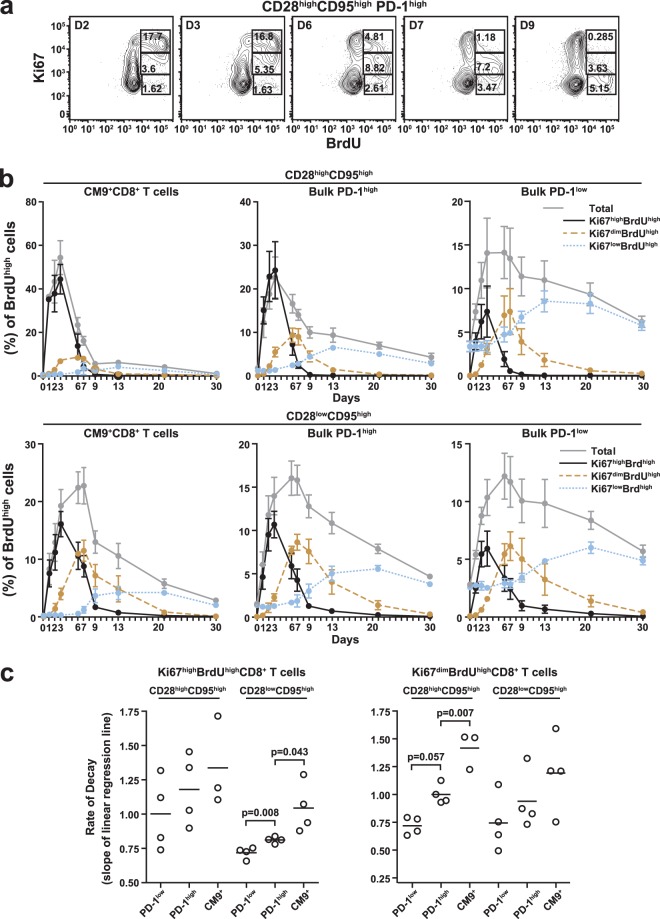
The majority of PD-1^high^ CD8 T cells do not return to a resting state. (a) Flow cytometry plots depicting the subpopulations of PD-1^high^ CD28^high^ CD95^high^ CD8 T cells identified by simultaneous analysis of Ki67 and *in vivo* integrated BrdU at several time points after BrdU administration. Numbers represent the relative frequency of the population in the box. (b) BrdU kinetics in total CM9^+^ and bulk memory CD8 T cell populations with stratification for Ki67 and BrdU levels. (Top) CD28^high^ CD95^high^ populations. (Bottom) CD28^low^ CD95^high^ populations. (c) BrdU decay rates in memory subsets of PD-1^low^, PD-1^high^, and CM9^+^ CD8 T cells with stratification for Ki67 expression. (Left) Ki67^high^ BrdU^high^ CD8 T cells. (Right) Ki67^dim^ BrdU^high^ CD8 T cells. The horizontal bars depict mean values, and the error bars indicate standard errors. *P* values were calculated using the Mann-Whitney *U* test.

Next, we analyzed the *in vivo* kinetics of individual CD8 T cell populations according to the expression of Ki67, an activation/proliferation marker that is downregulated when cells return to a resting state. The majority of CM9^+^ cells displayed a Ki67^high^ phenotype in both the CD28^high^ CD95^high^ (79.69 ± 1.78) and CD28^low^ CD95^high^ (52.78 ± 1.28) populations (see Fig. S1B in the supplemental material). Among BrdU^+^ cells, coexpression of Ki67 can be divided into three populations (Ki67^high^ BrdU^high^, Ki67^dim^ BrdU^high^, and Ki67^low^ BrdU^high^) and tracked over time (Fig. 3a). Analyzing the *in vivo* BrdU label contents in these populations for both CM9^+^ and total CD8 T cells allowed us to follow the kinetics of activation and return to rest. In the CM9^+^ CD28^high^ CD95^high^ population, BrdU peaked at day 3 or 4 in the Ki67^high^ BrdU^high^ state, followed by the Ki67^dim^ BrdU^high^ (peak at day 6 or 7) and Ki67^low^ BrdU^high^ (peak at days 13 to 20) populations (Fig. 3b, top). It is noteworthy, however, that these state transitions do not take place in the peripheral blood, where cells appear only very transiently; rather, the blood samples provide a window into the kinetics that take place in the lymph nodes. Importantly, more cells expressed the Ki67^high^ BrdU^high^ phenotype than progressed to the subsequent populations with lower levels of Ki67 expression (Fig. 3b, top). This phenomenon was more prominent in the CM9^+^ CD28^high^ CD95^high^ population than in the CM9^+^ CD28^low^ CD95^high^ population (Fig. 3b, bottom), thereby confirming our computational analysis, which had indicated a clear hierarchy in the ability of cells to return to rest after activation (Fig. 2b). Analysis of bulk CD8 T cells revealed a similar loss (through death or migration into the tissues) of cells during transition from an activated to a resting state in the PD-1^high^ compartment, especially in the CD28^high^ CD95^high^ population (Fig. 3b). Interestingly, this was not the case for bulk CD8 T cells expressing a PD-1^low^ phenotype, where BrdU^high^ cells transitioned through the phases of Ki67 expression without substantial loss in total cell numbers (Fig. 3b). Next, the BrdU decay rates were calculated for CD8 T cell populations in the Ki67^high^ BrdU^high^ and Ki67^dim^ BrdU^high^ compartment. Again, the CM9^+^ CD8 T cell population showed the highest decay rate among all populations tested, reaching statistical significance in the Ki67^high^ BrdU^high^ CD28^low^ CD95^high^ (0.811 ± 0.011 versus 1.044 ± 0.09 per day, bulk PD-1^high^ versus CM9^+^ CD8 T cells; *P* = 0.043) and Ki67^dim^ BrdU^high^ CD28^high^ CD95^high^ (1.001 ± 0.04 versus 1.417 ± 0.09 per day, bulk PD-1^high^ versus CM9^+^ CD8 T cells; *P* = 0.007) cellular compartments (Fig. 3c). Bulk PD-1^high^ CD8 T cells were consistently characterized by higher BrdU decay rates than bulk PD-1^low^ CD8 T cells in all populations tested (Fig. 3c), reflecting rapid proliferation-induced label dilution and replacement of labeled cells by unlabeled cells in the PD-1^high^ compartment.

### High turnover of CM9^+^ PD-1^high^ CD8 T cells during the acute phase of SIV infection.

**Fig 4:**
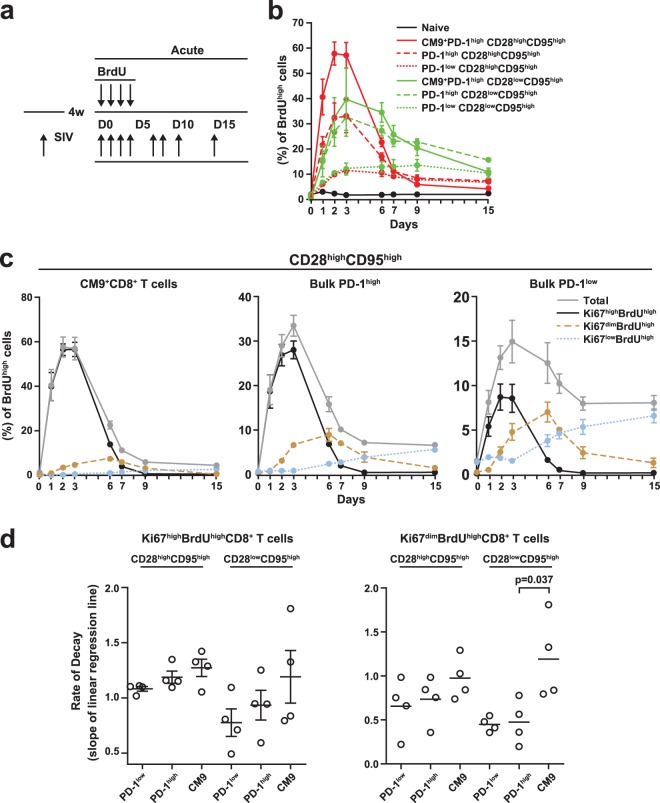
High *in vivo* turnover of CM9^+^ PD-1^high^ CD8 T cells during acute SIV infection. (a) Schematic representation of BrdU administration and blood collection during the acute phase. (b) BrdU kinetics in CM9^+^ and bulk memory CD8 T cells during the acute phase with stratification for PD-1 expression. (c) BrdU kinetics in CM9^+^ (left) and bulk memory (middle and right) CD8 T cells during the acute phase with stratification for Ki67 and BrdU levels. (d) BrdU decay rates in memory subsets of PD-1^low^, PD-1^high^, and CM9^+^ CD8 T cells during the acute phase with stratification for Ki67 expression. (Left) Ki67^high^ BrdU^high^ CD8 T cells. (Right) Ki67^dim^ BrdU^high^ CD8 T cells. The horizontal bars depict mean values, and the error bars indicate standard errors. *P* values were calculated using the Mann-Whitney *U* test.

In further experiments, we investigated the *in vivo* dynamics of BrdU incorporation 4 weeks after SIVmac251 infection (Fig. 4a). A low, transient integration of BrdU was observed in the CD28^dim^ CD95^low^ CD8 T cell population (Fig. 4b). Similar to the chronic phase of SIV infection, BrdU incorporation was highest in the CM9^+^ compartments of both memory CD8 T cell subsets (Fig. 4b; see Fig. S3 in the supplemental material); furthermore, BrdU loss was accelerated in the CM9^+^ population compared to matched bulk memory CD8 T cells (Fig. 4b). In contrast to the situation in chronic SIV infection, however, bulk PD-1^high^ CD28^high^ CD95^high^ and PD-1^high^ CD28^low^ CD95^high^ CD8 T cells showed similar maximum levels of BrdU incorporation (33 ± 6.6 versus 32.7 ± 7.6, maximum percentage of BrdU^high^ cells) (Fig. 4b). BrdU decay rates were significantly higher in CM9^+^ CD8 T cells (0.496 ± 0.05) than in bulk CD8 T cells in both the CD28^high^ CD95^high^ (0.183 ± 0.03 per day; *P* = 0.004) and CD28^low^ CD95^high^ (0.195 ± 0.01 per day; *P* = 0.002) compartments. Additionally, the PD-1^low^ population displayed lower decay rates than the PD-1^high^ population in both the CD28^high^ CD95^high^ (0.143 ± 0.03 versus 0.368 ± 0.04 per day; *P* = 0.004) and CD28^low^ CD95^high^ (0.094 ± 0.017 versus 0.185 ± 0.039 per day; *P* = 0.07) subsets. As in the chronic phase, simultaneous analysis of BrdU and Ki67 expression revealed complex BrdU dynamics in both the CM9^+^ and bulk CD8 T cell populations (Fig. 4c). A large difference in maximum BrdU incorporation was apparent between Ki67^high^ and Ki67^dim^ CM9^+^ cells (Fig. 4c), indicative of the fact that only a small fraction of such cells return to rest after activation. This profile was apparent for both the CD28^high^ CD95^high^ (56.78 ± 3.19 versus 7.41 ± 1.3, Ki67^high^ BrdU^high^ versus Ki67^dim^ BrdU^high^; *P* < 0.0001) and CD28^low^ CD95^high^ (34.35 ± 7.74 versus 16.43 ± 2.19, Ki67^high^ BrdU^high^ versus Ki67^dim^ BrdU^high^; *P* = 0.06) populations (Fig. 4c and data not shown). Again, CM9^+^ CD8 T cells showed the highest decay rates of all populations tested, even during acute infection (Fig. 4d). Therefore, the *in vivo* dynamic profile of CD8 T cell populations during the acute phase is similar to that observed in the chronic phase of SIV infection.

## DISCUSSION

Recently published work has shown that PD-1 could serve as a regulator of antigen-specific CD8 T cell survival (9, 21, 22). In particular, the level of PD-1 expression correlates with *in vitro* sensitivity to cell death in both HIV (9) and SIV (6) infection. This *in vitro* phenotype raises the question of what cellular mechanism(s) supports the sustained PD-1^high^ HIV/SIV-specific CD8 T cell populations observed *in vivo*. We considered two possibilities. First, the rapid disappearance of PD-1^high^ virus-specific CD8 T cells may be counterbalanced by rapid production (high generation/high disappearance). Second, PD-1^high^ CD8 T cells may not be as susceptible to apoptosis *in vivo* as they are *in vitro*, in which case a low generation/low disappearance rate could sustain PD-1^high^ virus-specific CD8^+^ T cells. The current study was designed to investigate the relative impacts of these mechanisms in mediating the potential proapoptotic function of PD-1. Accordingly, the *in vivo* turnover rates of several rigorously defined CD8 T cell populations with respect to PD-1 expression were determined in SIV-infected rhesus macaques.

Several previous studies have shown a significantly increased *in vivo* turnover of CD8 T cells in SIV-infected monkeys compared to noninfected animals (12, 14, 17). Furthermore, memory CD8 T cells were found to express a phenotype characterized by accelerated *in vivo* accumulation of BrdU, followed by rapid loss compared to naive CD8 T cells (12, 17), indicative of increased proliferation/disappearance of the former cells. In our study, no BrdU integration was found in the “naive” CD28^dim^ CD95^low^ CD8 T cell compartment during the chronic phase, and only transient BrdU integration in a small fraction of these cells was observed during the acute phase. Furthermore, only very low levels (<1%) of BrdU incorporation were detected before SIV infection, even in the PD-1^high^ bulk memory CD8 T cell compartment. This finding is in line with the very low levels of Ki67 expression previously reported in this population of cells in non-SIV-infected animals (23) and indicates that PD-1 does not demarcate proliferation by itself. Furthermore, the short time window (4 days) of BrdU administration in this study likely only permits the detection of cell populations with high turnover rates, in contrast to previous studies in which BrdU was given for 2 to 4 weeks (12, 14, 17). As expected, SIV-specific CD8 T cells were characterized by higher turnover rates than bulk CD8 T cells in both memory compartments tested. Additionally, high expression of PD-1 was consistently associated with higher *in vivo* turnover in all populations tested. This was more prominent in the less differentiated CD28^high^ CD95^high^ population than in the CD28^low^ CD95^high^ population for both total and SIV-specific CD8 T cells. The kinetics of BrdU integration were also found to be different in these two memory subsets, but only in the PD-1^high^ compartment. Thus, PD-1^high^ CD28^high^ CD95^high^ cells accumulated BrdU faster and reached maximum integration earlier than PD-1^high^ CD28^low^ CD95^high^ cells. Although the peak of BrdU integration was different, similar kinetics applied to SIV-specific and bulk CD8 T cells in the same memory compartment. These findings indicate that the expression of coinhibitory receptors, such as PD-1, as well as differentiation status, plays an important role in the regulation of CD8 T cell proliferation/disappearance *in vivo*.

The relative representation of phenotypically distinct CD8 T cell populations was found to be quite constant during the period of investigation in both the chronic and acute phases of SIV infection. An important aspect of this profile relates to whether a cell that starts out in one phenotype can be assumed to end in another phenotype. To explore this issue further, we would require far more data, because the models would include parameters for phenotypic transitions (differentiation steps). As our model does not allow for such transitions, we assumed the presence of a population that can receive a certain degree of stimulation and appear as PD-1^+^ in the blood. The data and the model show that most of these cells received a potent stimulus and rapidly underwent several divisions, after which they quickly disappeared. In agreement with our previous data (6), the vast majority of SIV-specific CD8 T cells expressed a PD-1^high^ phenotype during the chronic phase. This profile was associated with significantly higher Ki67 expression than in bulk PD-1^high^ CD8 T cells in both memory compartments tested, while PD-1^low^ cells expressed the lowest levels of Ki67. The application of a mathematical model revealed significantly higher generation rates in the SIV-specific CD8 T cell compartment than in bulk PD-1^high^ populations; PD-1^low^ cells displayed the lowest generation rates. Expression of Ki67, a marker of CD8 T cell activation, therefore, correlates with the *in vivo* turnover of total and virus-specific CD8 T cells during chronic SIV infection. This finding agrees with previously reported data from studies of HIV infection (15).

In the case of SIV-specific CD8 T cells, the loss of BrdU was found to follow a biphasic mode, with a rapid decline (before day 9) followed by a slower phase (after day 9). Such a biphasic mode was previously reported in other studies of HIV (13) and SIV (24) infection. The loss of BrdU could be due to cell death, migration to different anatomical compartments, and/or dilution in cells that continue to divide. Loss of Ki67 expression by BrdU-labeled cells after discontinuation of BrdU administration identifies cells that have stopped dividing, while the persistence of a population of cells with high levels of Ki67 expression but diminishing BrdU intensity helps to identify cells that continue to divide (16, 24). We therefore investigated the *in vivo* turnover of CD8 T cell populations further by analyzing the kinetics of cells coexpressing BrdU and Ki67. BrdU dynamics were found to be dramatically different between SIV-specific CD8 T cell populations with respect to Ki67 expression. A hierarchy was observed both for peak height and the rate of decline of BrdU in the different populations tested. The Ki67^high^ BrdU^high^ population exhibited the highest integration/decline rate of BrdU, followed by the Ki67^dim^ BrdU^high^ and Ki67^low^ BrdU^high^ populations. This profile was more prominent in the less differentiated CD28^high^ CD95^high^ memory cell subset. A similar profile was observed in the bulk PD-1^high^ compartment. In the PD-1^low^ compartment, all three populations (Ki67^high^ BrdU^high^, Ki67^dim^ BrdU^high^, and Ki67^low^ BrdU^high^) exhibited similar peak heights. Label loss was faster in the PD-1^high^ than in the PD-1^low^ compartment regardless of Ki67 expression, supporting a potential proapoptotic role for PD-1 in cells expressing a PD-1^high^ phenotype. Furthermore, the slower decline of PD-1^high^ BrdU-labeled cells in the Ki67^dim^ and Ki67^low^ populations than in the Ki67^high^ population likely stems from a greater disappearance of BrdU^high^ cells in the PD-1^high^ than in the PD-1^low^ compartment. This conclusion is further supported by the results of our computational analysis, which indicates a considerably lower rate of return to rest for PD-1^high^ cells.

PD-1 is a marker of potent stimulation and demarcates cells that have received proproliferative signals. In the experimental data, we cannot distinguish cells that received these stimuli over a prolonged period of time, performed several divisions, and then assumed a state that corresponds to desensitization from cells that simply upregulated PD-1 due to recent stimulation, thereby retaining the ability to undergo rapid division pending receipt of strong inhibitory signals via PD-1. For the modeling process, however, this lack of distinction was not critical, because we only assumed the existence of a population of cells that can receive a certain degree of stimulation and appear as PD-1^high^ in the blood. Again, our data show that the vast majority of PD-1^high^ cells received strong stimulation, rapidly underwent several divisions, and then disappeared.

Our simple computational analysis assumed a self-contained PD-1^high^ population whose homeostasis is maintained by a (small) fraction of cells returning to rest after activation and proliferation. Reconciling our findings of vigorous proliferation with a previously assumed role of PD-1 as a marker of defective responsiveness to antigenic stimulation, one could hypothesize that PD-1^high^ T cells, after leaving a proliferative state, do not return to a pool of cells that can be restimulated to undergo further rounds of division. Thus, the virus-specific Ki67^−^ BrdU^+^ PD-1^high^ cells observed in our study might not be contributing to the long-term persistence of the PD-1^high^ phenotype. One possibility is that new PD-1^high^ virus-specific CD8 T cells are continuously generated, especially in the setting of chronic infection. Alternatively, PD-1^low^ cells could continuously convert to a PD-1^high^ phenotype by upregulating PD-1 upon stimulation/activation. A more finely resolved kinetic analysis of BrdU incorporation and phenotypic transition will be necessary to distinguish between these possibilities.

## Supplementary Material

Supplemental material
