# Supplementary material for: High Production Rates Sustain In Vivo Levels of PD-1high Simian Immunodeficiency Virus-Specific CD8 T Cells in the Face of Rapid Clearance
Source: J Virol. 2013 Sep;87(17):9836–44. doi: 10.1128/JVI.01001-13 (PMC3754085; doi:10.1128/JVI.01001-13)
Supplement: Supplemental material [file supp_87_17_9836__index.html]

High Production Rates Sustain In Vivo Levels of PD-1high Simian Immunodeficiency Virus-Specific CD8 T Cells in the Face of Rapid Clearance — Supplemental material 

# High Production Rates Sustain *In Vivo* Levels of PD-1high Simian Immunodeficiency Virus-Specific CD8 T Cells in the Face of Rapid Clearance

## Supplemental material

**Files in this Data Supplement:**

- Supplemental file 1 -

  Fig. S1A (Gating strategy for the detection of Ki67 and *in vivo* integrated BrdU in bulk and SIV-specific CD8 T cell populations.)

  Fig. S1B (SIV-specific CD8 T cell frequencies across the duration of the study as detected by CM9 and TL8 tetramer staining; percentages of PD-1high and Ki67high cells in CM9+ and bulk CD8 T cell populations in both memory compartments.)

  Fig. S2 (Fitting curves for BrdU levels in four different cell populations from three macaques.)

  Fig. S3 (BrdU kinetics in CD8 T cell populations during the acute and chronic phases of SIV infection for each macaque.)

  PDF, 1.3M
