# Supplementary material for: High Production Rates Sustain In Vivo Levels of PD-1high Simian Immunodeficiency Virus-Specific CD8 T Cells in the Face of Rapid Clearance
Source: J Virol. 2013 Sep;87(17):9836–44. doi: 10.1128/JVI.01001-13 (PMC3754085; doi:10.1128/JVI.01001-13)
Supplement: Supplemental material [file JVI.01001-13_zjv999098038so1.pdf]

Supplementary Figure Legends

Supplementary Figure S1. (A) Gating strategy for the detection of Ki67 and *in vivo* integrated BrdU in bulk and SIV-specific CD8 T cell populations. Memory subsets were identified based on the expression of CD28 and CD95. PD-1^{high} and PD-1^{low} populations were distinguished with reference to PD-1 expression on "naïve" (CD28^{dim}CD95^{low}) CD8 T cells. (B) SIV-specific CD8 T cell frequencies across the duration of the study as detected by CM9 and TL8 tetramer staining (upper left panel), and the percentage (%) of PD-1^{high} (upper right panel) and Ki67^{high} (lower panel) cells in CM9^{+} and bulk CD8 T cell populations in both memory compartments. The bars depict standard deviation values.

Supplementary Figure S2. Fitting curves for BrdU levels in the indicated cell populations from three macaques. The red data points and curve correspond to the total BrdU uptake; the blue data points and curve represent only the Ki67^{-}BrdU^{+} cells.

Supplementary Figure S3. BrdU kinetics in CD8 T cell populations during the acute and chronic phase of SIV infection for each macaque. Viral load (copies/ml) during the chronic phase is also shown.

Figure S1

a

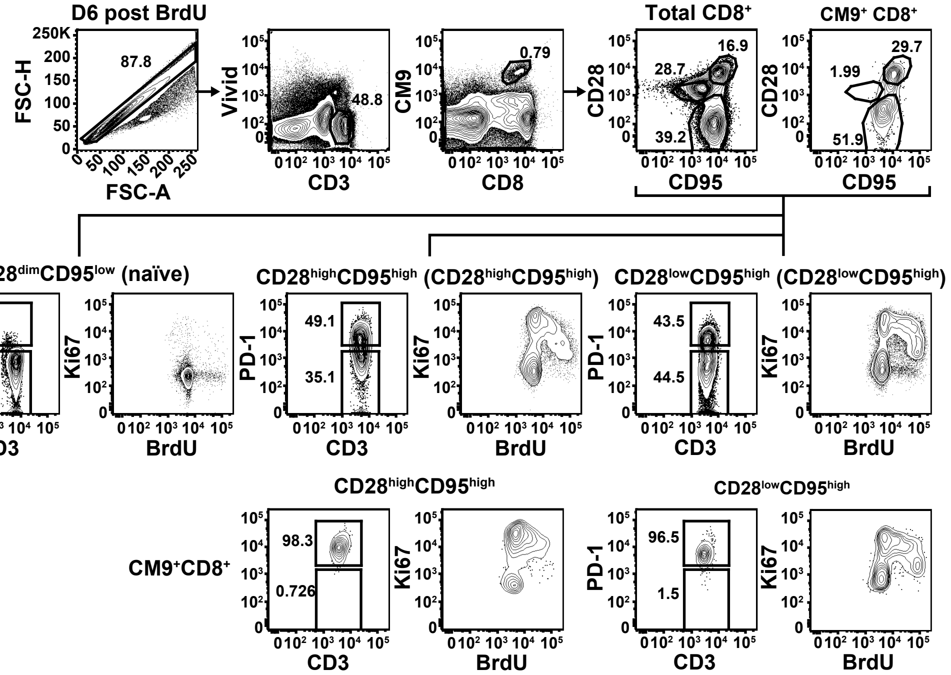
D6 post BrdU
FSC-H
FSC-A
87.8
Vivid
CD3
48.8
CM9
CD8
0.79
Total CD8⁺
CD28
CD95
28.7
16.9
39.2
CM9⁺ CD8⁺
1.99
29.7
51.9
CD28dimCD95low (naïve)
CD28highCD95high
(CD28highCD95high)
CD28lowCD95high
(CD28lowCD95high)
Bulk CD8⁺
PD-1
Ki67
BrdU
0.752
97.6
49.1
35.1
43.5
44.5
CM9⁺CD8⁺
98.3
0.726
96.5
1.5

b

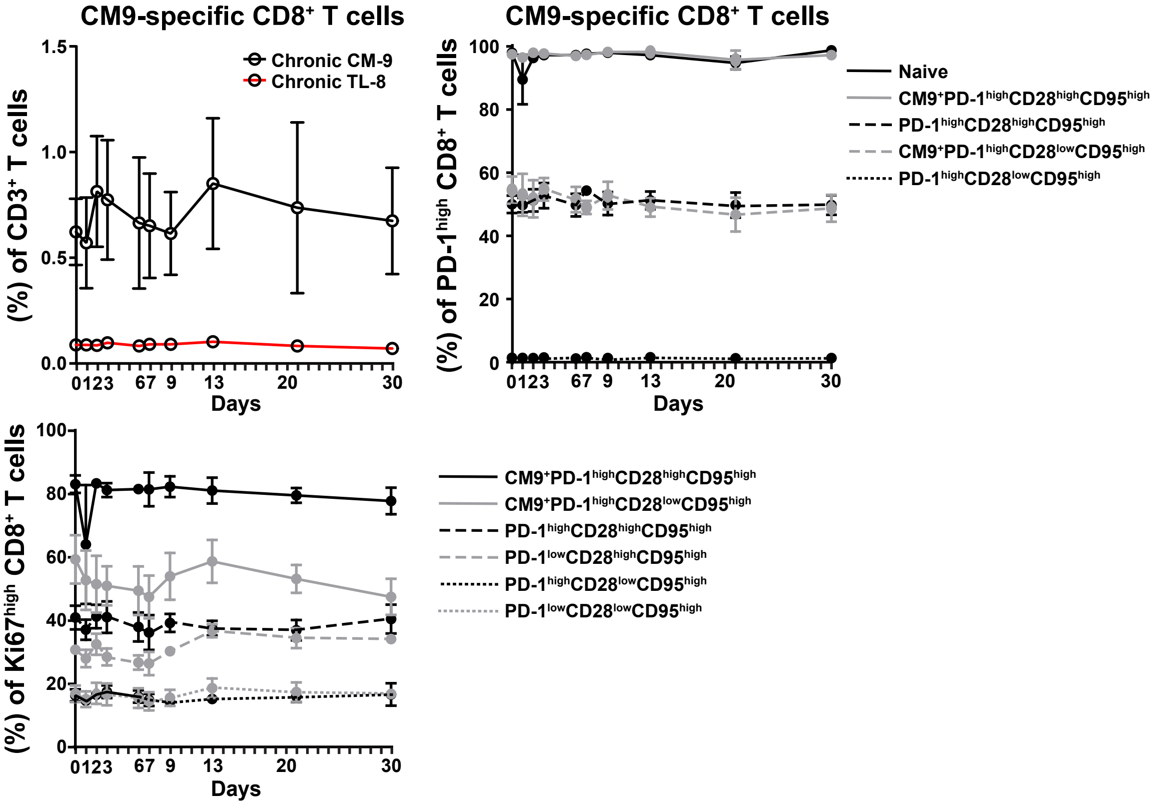
CM9-specific CD8⁺ T cells
(%) of CD3⁺ T cells
Chronic CM-9
Chronic TL-8
Days
(%) of PD-1high CD8⁺ T cells
Naive
CM9⁺PD-1highCD28highCD95high
PD-1highCD28highCD95high
CM9⁺PD-1highCD28lowCD95high
PD-1highCD28lowCD95high
(%) of Ki67high CD8⁺ T cells
CM9⁺PD-1highCD28highCD95high
CM9⁺PD-1highCD28lowCD95high
PD-1highCD28highCD95high
PD-1lowCD28highCD95high
PD-1highCD28lowCD95high
PD-1lowCD28lowCD95high

Figure S2

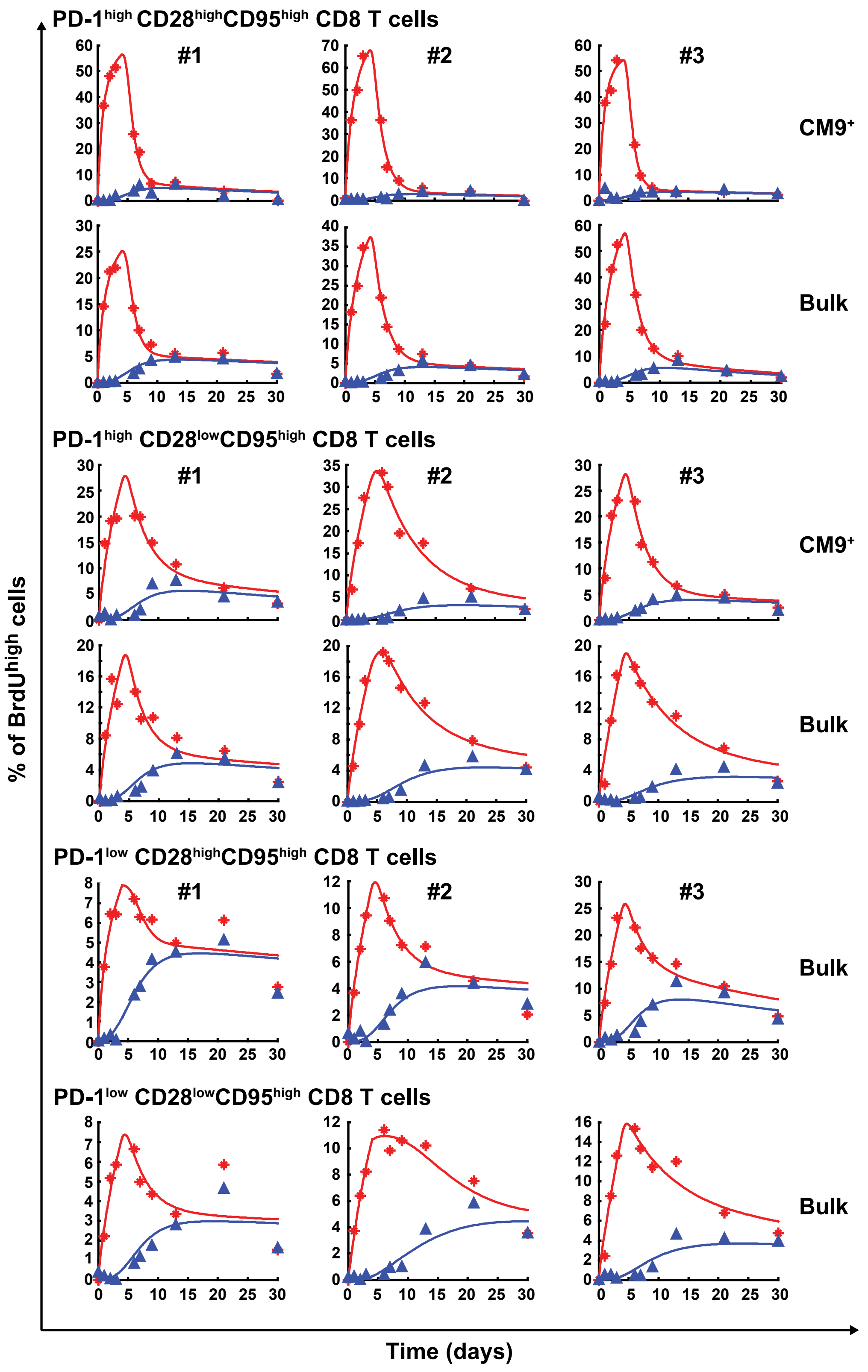

PD-1^high CD28^high CD95^high CD8 T cells
#1
#2
#3
CM9⁺
Bulk
PD-1^high CD28^low CD95^high CD8 T cells
PD-1^low CD28^high CD95^high CD8 T cells
PD-1^low CD28^low CD95^high CD8 T cells
% of BrdU^high cells
Time (days)

Figure S3

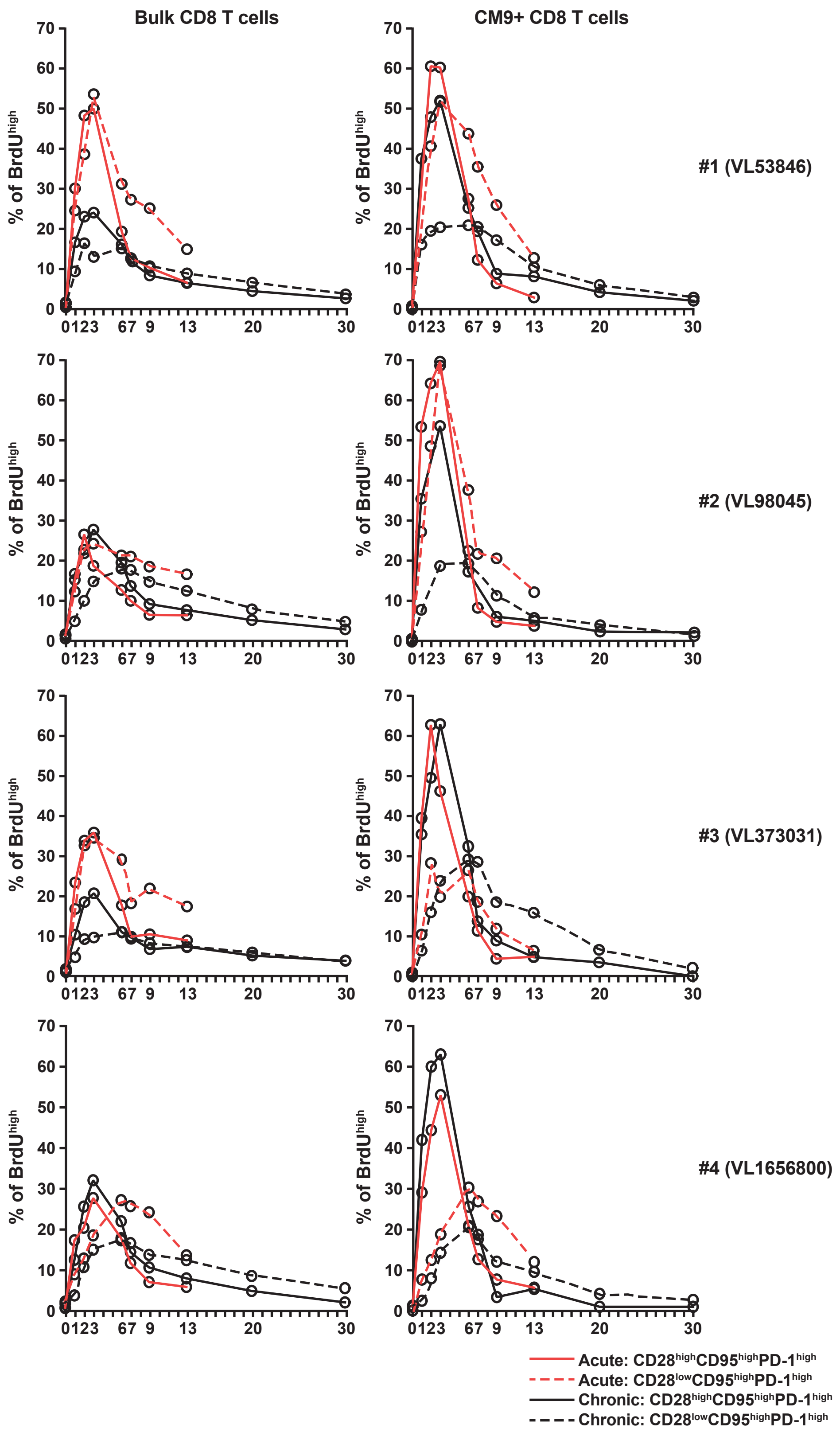

Bulk CD8 T cells
CM9+ CD8 T cells
% of BrdU^high
#1 (VL53846)
#2 (VL98045)
#3 (VL373031)
#4 (VL1656800)
0 10 20 30 40 50 60 70
0 1 2 3 6 7 9 13 20 30
Acute: CD28highCD95highPD-1high
Acute: CD28lowCD95highPD-1high
Chronic: CD28highCD95highPD-1high
Chronic: CD28lowCD95highPD-1high
